# Genome-Wide Identification and Expression Analysis of MADS-Box Family Genes in Litchi (*Litchi chinensis* Sonn.) and Their Involvement in Floral Sex Determination

**DOI:** 10.3390/plants10102142

**Published:** 2021-10-09

**Authors:** Hongling Guan, Han Wang, Jianjun Huang, Mingxin Liu, Ting Chen, Xiaozhen Shan, Houbin Chen, Jiyuan Shen

**Affiliations:** 1 Key Laboratory of Biology and Germplasm Enhancement of Horticultural Crops in South China, Ministry of Agriculture, South China Agricultural University, Guangzhou 510642, China; hlguan@stu.scau.edu.cn (H.G.); wanghan2019@stu.scau.edu.cn (H.W.); jjhuang@stu.scau.edu.cn (J.H.); lmx@stu.scau.edu.cn (M.L.); ct@stu.scau.edu.cn (T.C.); sxz1122@stu.scau.edu.cn (X.S.); 2 Guangdong Litchi Engineering Research Center, College of Horticulture, South China Agricultural University, Guangzhou 510642, China; 3 Maoming Branch, Guangdong Laboratory for Lingnan Modern Agriculture, Maoming 525000, China

**Keywords:** litchi, MADS-box genes, sex determination, flower development, phytohormones

## Abstract

Litchi possesses unique flower morphology and adaptive reproduction strategies. Although previous attention has been intensively devoted to the mechanisms underlying its floral induction, the molecular basis of flower sex determination remains largely unknown. MADS-box genes are promising candidates for this due to their significant roles in various aspects of inflorescence and flower organogenesis. Here, we present a detailed overview of phylogeny and expression profiles of 101 MADS-box genes that were identified in litchi. These *LcMADS*s are unevenly located across the 15 chromosomes and can be divided into type I and type II genes. Fifty type I MADS-box genes are subdivided into Mα, Mβ and Mγ subgroups, while fifty-one type II *LcMADS*s consist of 37 MIKC^C^ -type and 14 MIKC *-type genes. Promoters of both types of LcMADS genes contain mainly ABA and MeJA response elements. Tissue-specific and development-related expression analysis reveal that *LcMADS51* could be positively involved in litchi carpel formation, while six MADS-box genes, including *LcMADS42/46/47/75/93/100*, play a possible role in stamen development. GA is positively involved in the sex determination of litchi flowers by regulating the expression of *LcMADS51* (*LcSTK*). However, JA down-regulates the expression of floral organ identity genes, suggesting a negative role in litchi flower development.

## 1. Introduction

MADS-box family genes act as critical transcription factors in both reproductive and vegetative developments in plants [1]. The term ‘MADS’ was derived from the four earliest members of this family in fungi, plants and animals: *MCM1* from yeast, *AGAMOUS* (AG) from *Arabidopsis*, *DEFICIEN*S from snapdragon, and *SERUM RESPONSE FACTOR* (SRF) from humans [2,3,4,5]. All members of the family contain a highly conserved MADS-box motif, which encodes a 60 amino acid peptide responsible for nuclear localization, binding to the target DNA sequence [the CArG-box, CC(A/T) _6_ GG], and dimerization and binding of accessory factors [6].

In eukaryotes, the MADS-box gene family can be categorized into two groups, referred to as type I and type II [7]. In contrast to type I genes, which are weakly characterized in plants, type II subfamily genes have been extensively studied and documented. Type II proteins are also called MIKC-type proteins, named after the four characteristic domains: a MADS-box domain that determines DNA binding and dimerization of proteins; a less-conserved intervening (I) domain that is crucial to the formation of DNA dimers; a well-conserved keratin (K) domain that is involved in protein-protein interaction due to a coiled-coil structure, and a variable C-terminal (C) domain [6,7]. According to phylogenetic relationships, type I MADS-box genes can be further divided into Mα, Mβ, and Mγ classes [8], while type II genes can be subdivided into MIKC^C^ (the ^C^ means classic) and MIKC * classes based on structure differences in their K-domains and the length of their I domains. In comparison with MIKC *, the length of I domains is shorter and encoded by fewer exons in MIKC^C^ class proteins [9]. MIKC^C^-type MADS-box genes are further classified into approximately 11–13 clades based on structure differences and are well-known for their roles of significance in flower development according to the ABC model [10,11]. Thereafter, more important players of the floral process pathway were identified, leading to the extension of the ABC model to the ABCDE model and the protein-based quartet model [12]. 

Most angiosperm plants, such as *Arabidopsis*, consist of four sorts of typical flower organs, including sepals, petals, stamens, and carpels, arranged in a succession of concentric whorls or rings. The ABC model postulates three classes of genes that function in adjacent whorls. A-class genes *APETALA1* (*AP1*) and *APETALA2* (*AP2*) are involved in sepal and petal development; B-class genes *APETALA3* (*AP3*) and *PISTILLATA* (*PI*) specify the identity of petal and stamen; and C-class gene *AGAMOUS* (*AG*) is necessary for stamen and carpel specification. In addition, D-class genes *SHATTERPROOF* (*SHP*) and *SEEDSTICK* (*STK*) are found to determine ovule development, while E-class genes *SEPALLATA* (*SEP*) *1–4* assist in the formation of all floral organs [13,14,15]. All genes involved in the ABCDE model belong to the MADS-box gene family, except for *AP2*. 

Litchi is an economically valuable species in the Sapindaceae family, which is widely grown in southern China and subtropical regions due to its popular fruits. Litchi is a monoecious plant that produces determinate inflorescences on current-season terminal shoots. The litchi flowers possess a cup-shaped calyx with 4–5 sepals, but do not have petals. Based on the development and function of stamens and carpels, litchi flowers can be classified into three types, namely male flowers (type I), hermaphrodite functional female (type II) and hermaphrodite functional male flowers (type III). Type I flowers lack ovules and are functionally male (Male = M). These flowers have 6–8 stamens, which produce much viable pollen. Type II flowers are hermaphrodite but function as female (female = F), with a well-developed pistil (two carpels) and stigma (two-lobed), as well as stamens that do not dehisce. Type III flowers are male (male = m) but have a rudimentary pistil lacking style and stigma. In general, the three types of flowers bloom in the succession of Male-female-male, namely the first flowers to open are male flowers (type I), whereas in some particular years the first flowers to open can be female [16], in which we hypothesize that genes controlling carpel development are activated and promoted ahead of time. However, little is known about the molecular basis and regulation mechanisms underlying the sex differentiation of litchi flowers. 

In this study, MADS-box genes in litchi were identified because of their versatile roles in flowering and flower development, and their chromosomic locations were mapped. Gene structures, conserved motifs, and cis-elements of their promoters were comprehensively analyzed. The tissue-specific expression profiles of MADS-box genes in stamens and carpels of three types of flowers at different stages was investigated. In addition, candidate genes for flower sex determination in response to gibberellin (GA) and methyljasmonate (MeJA) were evaluated. The results can improve our understanding of the evolution and functions of MADS-box genes in litchi and facilitate further studies of molecular mechanisms in the sex determination of litchi flowers.

## 2. Results

### 2.1. Identification and Characterization of MADS-Box Genes in Litchi

**Figure 1:**
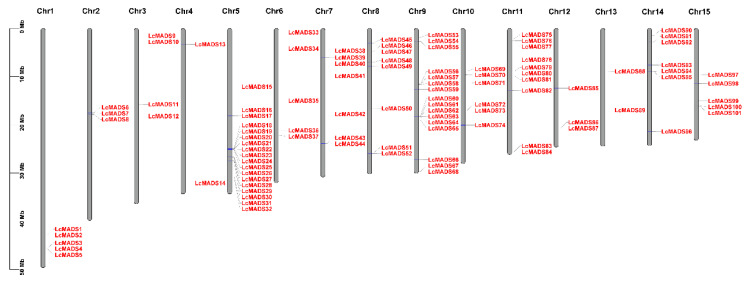
Schematic representations of the chromosomal location of litchi MADS-box genes. The chromosome number is indicated at the top of each chromosome.

To identify candidate MADS-box genes in litchi, BLASTP was conducted to search the litchi genome database using MADS-box protein sequences in *Arabidopsis*, rice, and tomato as queries. The full-length coding sequence (CDS) of 154 putative MADS-box genes was obtained. In addition, a Hidden Markov Model (HMM) search in the litchi genome was carried out using the SRF-TF domain (PF00319), resulting in a total of 199 putative MADS-box genes. Subsequently, these sequences were further verified through blasting against public databases, including Conserved Domain Database (CDD) and Simple Modular Architecture Research Tool (SMART). The sequences without conserved MADS-box domains and alternative transcripts were removed, resulting in a final total of 101 sequences identified as MADS-box genes in litchi (Figure 1). All 101 genes were unevenly distributed on 15 chromosomes in litchi and were named as *LcMADS1* to *LcMADS101*, based on their position on the chromosome (Figure 1). Chr5 had the greatest number of MADS-box genes (18 genes), followed by Chr9 (16 genes), while Chr4 and Chr13 had only 2 genes each (Figure 1). 

The CDS length of litchi MADS-box genes ranged from 228 bp (*LcMADS16*) to 1317 bp (*LcMADS98*). Accordingly, the relative molecular weight (MW) varied from 8.44 kDa to 48.94 kDa, and the theoretical pI ranged from 5.16 to 10.37 (Table S1). The diversity in the amino acid sequence length, MW, and pI of LcMADSs indicated functional differences between them.

### 2.2. Phylogenetic Analysis of Litchi MADS-Box Genes

**Figure 2:**
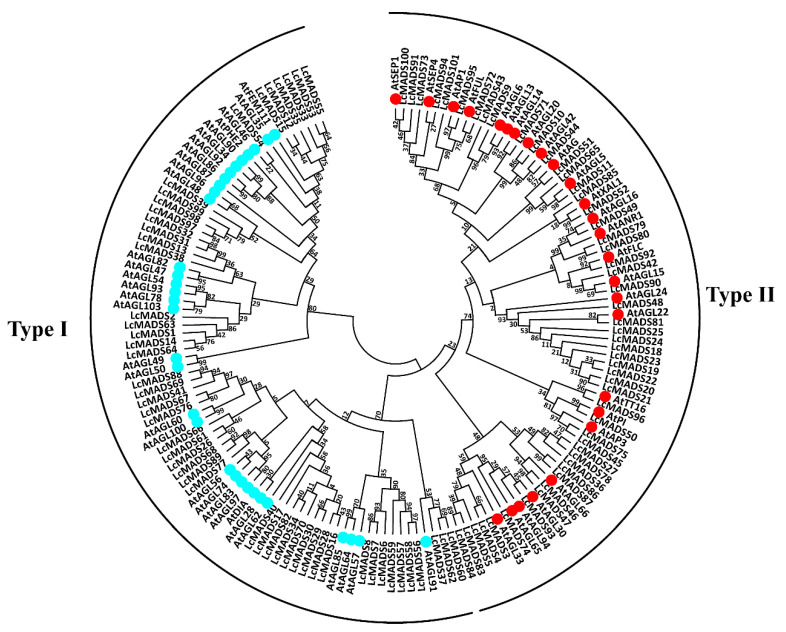
Phylogenetic analysis of litchi and *Arabidopsis* MADS-box proteins. A total of 58 representative MADS-box proteins from different subfamilies of *Arabidopsis* were used to construct the phylogenetic tree by the maximum likelihood method. These MADS-box factors are classified into two clades, designated as type I and type II.

In order to investigate the evolutionary relationship between litchi MADS-box genes (101 genes) and the known MADS-box genes in *Arabidopsis* (58 genes), we conducted phylogenetic analysis based on multiple alignment of full-length protein sequences. According to the maximum likelihood phylogenetic tree, 50 litchi MADS-box proteins were clustered into type I, and the remaining 51 proteins were classified into type II (Figure 2).

**Figure 3:**
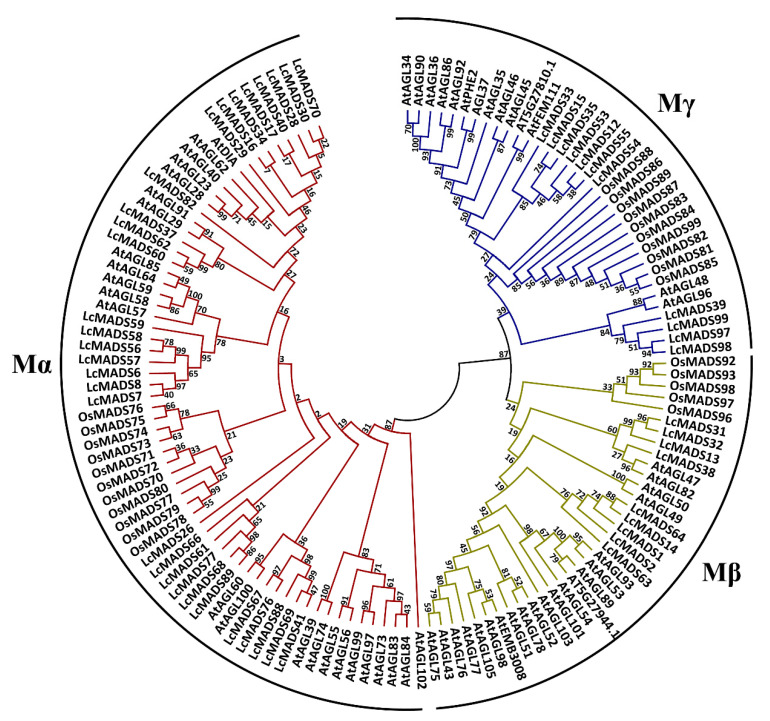

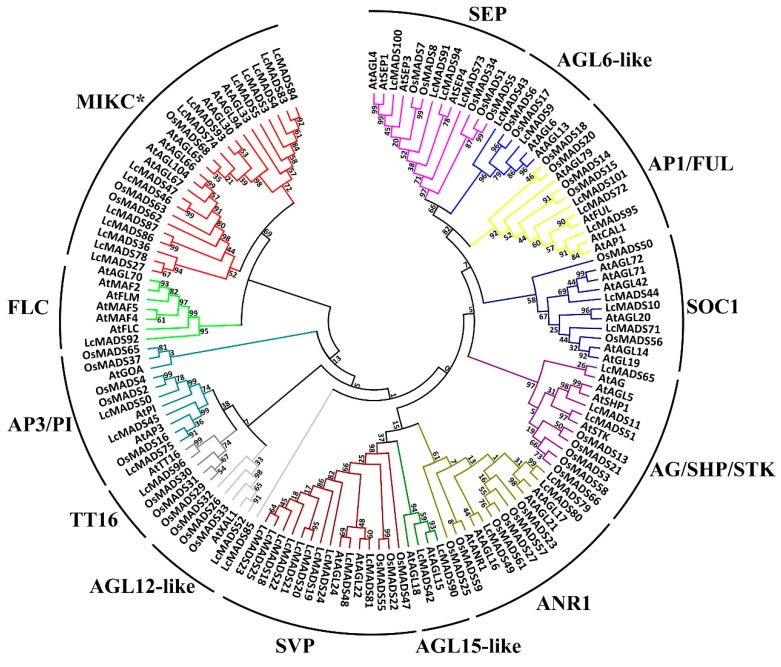
Phylogenetic relationship analysis of type I (top) and type II (bottom) MADS-box transcription factors in litchi, *Arabidopsis*, and rice, based on construction of a maximum likelihood tree. Type I members were grouped into 3 subfamilies, while type II members were grouped into 13 subfamilies as indicated by different branch colors. Abbreviations: ANR1: ANTHOCYANIDIN REDUCTASE 1; FUL: FRUITFULL; TT16: TRANSPARENT TESTA 16.

To further examine the phylogenetic relationship between litchi MADS proteins and group them into the established subfamilies, we performed phylogenetic analysis separately for type I and type II from alignments of full-length protein sequences from litchi, *Arabidopsis*, and rice by the maximum likelihood method. According to the phylogenetic tree, fifty type I MADS-box genes were divided into three subfamilies, including Mα (30 genes), Mβ (9 genes), and Mγ (11 genes) (Figure 3). Fifty-one type II MADS-box genes could be divided into 13 subfamilies, including 14 MIKC *-type genes and 37 MIKC^c^-type genes (Figure 3). In general, litchi and *Arabidopsis* have a similar number of genes in each subfamily, except for the SVP (SHORT VEGETATIVE PHASE) subfamily, in which litchi had 10 members while *Arabidopsis* and rice had only 2 and 3 members, respectively (Figure 3). In contrast to the SVP subfamily, *Arabidopsis* had more members in the FLC (FLOWERING LOCUS C) subfamily than litchi, with rice possessing no FLC homologs (Figure 3).

### 2.3. Identification of Gene Domain, Structure, and Conserved Motif 

Generally, type I proteins have only a MADS domain, while type II MADS proteins contain both MADS and K domains. In litchi, 65 MADS proteins have only MADS domains, while 36 have both MADS and K domains, according to SMART and CDD analysis. Interestingly, 15 out of 51 type II litchi MADS-box genes, similar to type I genes, lacked the K domain. The 14 non-K domain genes were located in the MIKC * subfamily, and the remaining one belonged to the SVP subfamily of MIKC^C^ (Table S1 and Figure S2).

**Figure 4:**
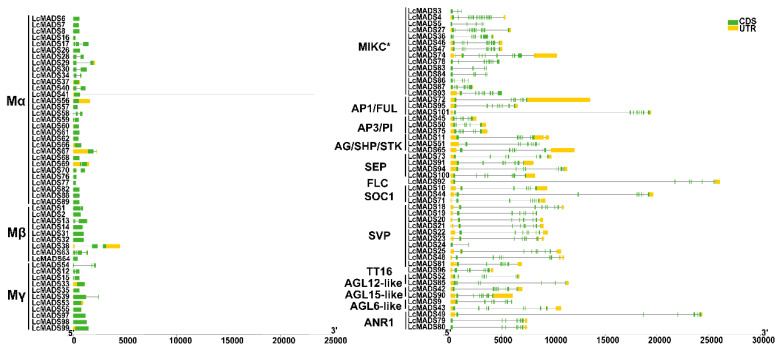
Structural analysis of MADS-box transcription factor genes in litchi. Yellow boxes indicate untranslated 5′- and 3′-regions, while green boxes and black lines indicate exons and introns, respectively. Type I genes are shown on the left and type II genes are shown on the right.

**Figure 5:**
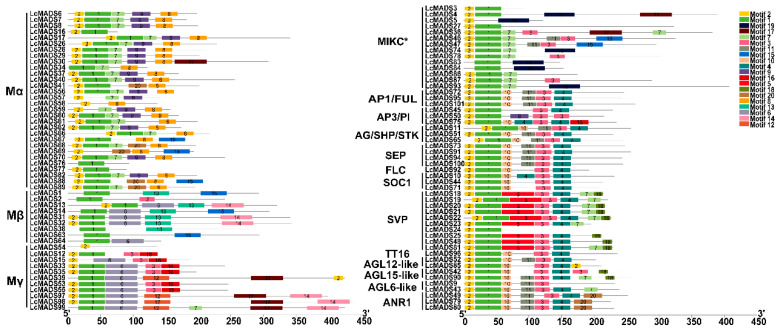
Conserved motif composition of litchi MADS-box proteins. The motifs, numbered 1–20, are indicated with colored boxes. The specific motif information is provided in Table S2. Type I genes are shown on the left and type II genes are shown on the right.

The analysis of intron-exon organization showed that type II genes contained more introns compared to type I genes, with the MIKC * genes containing the largest number of introns (Figure 4). Genes in the same group were likely to have a similar number of introns and exons. However, some closely clustered genes within a subfamily showed significant differences in gene structural arrangement. For example, *LcMADS24* in the SVP subfamily possessed only 2 exons, while other closely related genes in this subfamily had 6 or 7 exons (Figure 4). Furthermore, 20 conserved motifs within the 101 litchi MADS genes were predicted using the MEME motif search tool (Figure 5). The lengths of 20 conserved motifs ranged from 15 to 50 amino acids (Table S2). Motif 1 and 2 represent MADS domains, while motif 3 and 4 are two fragments of the K domain. All litchi MADS-box genes, except for *LcMADS5*, *15*, *57*, *58*, *59* and *69,* contained motif 1, and the six genes without motif 1 possessed motif 2. Motif 3 and 4 were identified in the majority of type II MADS-box genes, whereas they were present only in seven type I genes (*LcMADS2*, *12*, *15*, *33*, *35*, *53* and *55*) (Figure 5).

### 2.4. Expression Profiles of Litchi MADS-Box Genes in Different Floral Organ Tissues

**Figure 6:**
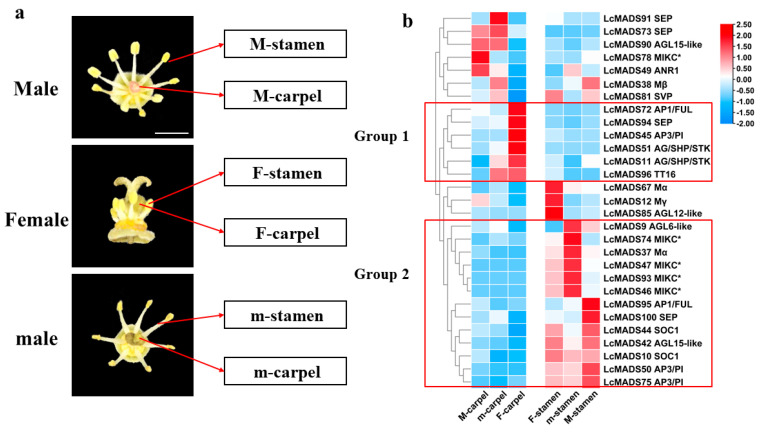
Expression profiles of litchi MADS-box genes in various floral organs. M-carpel, the carpels in full-bloom functionally male flowers (Male, M); M-stamen, the stamens in full-bloom functionally male flowers (Male, M); F-carpel, the carpels in full-bloom functionally female flowers (Female, F); F-stamen, the stamens in full-bloom functionally female flowers (Female, F); m-carpel, the carpels in full-bloom functionally male flowers (male, m); m-stamen, the stamens in full-bloom functionally male flowers (male, m) (**a**). FPKM values of litchi MADS-box genes derived from the transcriptome data were normalized to z-scores for each row to construct the heat map (**b**). Scaled log_2_ expression values are shown from blue to red, indicating low to high expression level.

In order to investigate the role of MADS-box genes in litchi floral organ determination, the expression patterns of these genes were analyzed based on transcriptome data and real-time PCR-based expression analysis. Three types of floral organ tissues, including full-bloom functionally female flowers with rudimentary stamens (Female, F), functionally male flowers with rudimentary unobvious pistils (Male, M), and functionally male flowers with rudimentary obvious pistils (male, m), were collected (Figure 6a). Detached carpels and stamens were separately sampled for RNA extraction, and FPKM (fragments per kilobase of transcript per million mapped) values of MADS-box genes were calculated to obtain the differentially expressed *LcMADS* genes. A total of 29 differentially expressed *LcMADS*s were filtered based on pairwise comparisons of FPKM values (Fold change ≥ 2 and FDR < 0.01), and the data were visualized using a heat map (Figure S3). 

Among the differentially expressed genes, two groups can be distinguished, differing in the character of expression in the organs of flowers of different sexual functionality. Based on the expression trend, genes in group I were highly correlated with carpel development, while genes in group II were highly correlated with stamen development (Figure 6b). Six genes in group I (*LcMADS11*, *45*, *51*, *72*, *94* and *96*) were highly expressed in the carpel of functionally female flowers but showed less mRNA abundance in stamens and the carpel of functionally male flowers. In contrast, all genes in group II showed a much higher expression level in stamens than carpels. The differentially expressed genes of both type I and II outside the two groups showed no/negative significant correlation with flower sex differentiation. For example, *LcMADS12/67/85* showed the highest expression level in F-stamens, whereas stamens were highly under-developed in litchi female flowers, implicating a negative correlation with stamen development. 

### 2.5. Gene Expression of Litchi MADS-Box Genes during Flower Development

**Figure 7:**
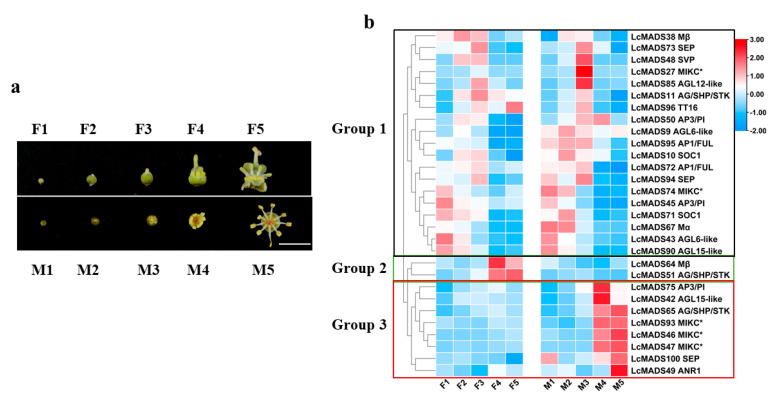
Expression profiles of litchi MADS-box genes at different stages of flower development. Fl, 0.5–1 mm female flower buds; F2, 1–1.5 mm female flower buds; F3, 1.5–2 mm female flower buds; F4, half-bloom female flowers; F5, full-bloom female flowers; M1, 0.5–1 mm male flower buds; M2, 1–1.5 mm male flower buds; M3, 1.5–2 mm male flower buds; M4, half-bloom male flowers; M5, full-bloom male flowers (**a**). FPKM values of litchi MADS-box genes from the transcriptome data were normalized to z-scores for each row to construct the heat map (**b**).

To explore the role of MADS-box genes in flower development, we studied the expression pattern of MADS-box genes in different stages of female and male flower development (Figure 7a). The genes without significant differential expression (Fold change ≥ 2 and FDR < 0.01) were filtered out (Figure S4). According to the expression pattern, genes in group I showing a higher expression level at the early stages of male or female flower development might be negatively associated with litchi flower development or play a role during the early developmental stages of litchi flowers. On the contrary, the expression of genes in group II and III increased as male or female flowers developed, respectively, indicating a positive role in flower determination and development (Figure 7b). Based on tissue-specific and development-related expression analysis, *LcMADS51* could be positively involved in litchi carpel formation, while six MADS-box genes, including *LcMADS42*/*46*/*47*/*75*/*93*/*100*, play a possible role in stamen development (Figure 6 and Figure 7). 

Four MADS-box genes were randomly selected for Q-PCR analysis in different flower tissues and during flower development to verify the RNA-seq data (Figures S5–S7). The relative expression of these four genes was highly consistent with their FPKM value, derived from RNA-Seq libraries, indicating that the FPKM value could well represent the expression level of genes. 

### 2.6. Prediction of Promoter Elements in Litchi MADS-Box Genes

Plant hormones play important roles in litchi flowering and flower development. In order to further understand the response of litchi MADS-box genes to phytohormones, cis-acting element analysis was performed using the promoter region (2-kbupstream space transcription start codon) of all 101 genes. Cis-elements were classified into six broad categories based on their responses to plant hormones, namely auxin, abscisic acid (ABA), ethylene, gibberellin (GA), methyl jasmonate (MeJA), and salicylic acid (SA).

**Figure 8:**
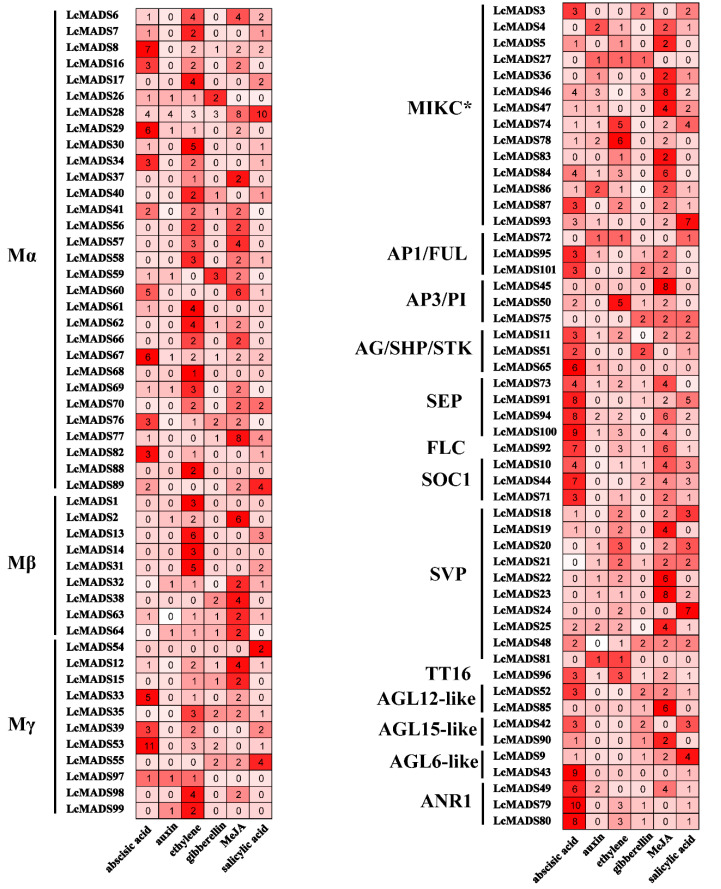
Predicted cis-elements in promoters of litchi MADS-box genes using PlantCARE. The numbers of abscisic acid, auxin, gibberellin, MeJA, salicylic acid, and ethylene related cis-elements are indicated. Type I genes are shown on the left and type II genes are shown on the right.

Type I genes contained up to eleven ABA response elements, up to six ethylene response elements, and up to eight MeJA response elements, while Type II genes possessed up to ten ABA response elements and up to eight MeJA response elements (Figure 8, Table S4). AG/SHP/STK, SEP, FLC, SOC1 (SUPPRESSOR OF OVEREXPRESSION OF CONSTANS1), and ANR1 subgroups had the most ABA response elements (Figure 8). For example, *LcMADS24* and *LcMADS93* promoter sequences contained up to seven salicylic acid response elements, suggesting these genes are strongly responsive to salicylic acid signals. Many members contained ethylene response elements, such as *LcMADS30* in subfamily Mα, *LcMADS14* in subfamily Mβ, *LcMADS78* in subfamily MIKC *, and *LcMADS50* in subfamily AP3/PI. However, auxin and GA response elements were rarely present in the promoter of MADS-box genes (Figure 8).

### 2.7. Expression of MADS-Box Genes in Response to the Treatment of Hormones

To validate the response of *LcMADS* to hormones, we analyzed the expression of putative ABCDE genes in inflorescences at different times after hormone treatments. According to the phylogenetic relationship analysis (Figure 3), *LcMADS95* (*LcAP1*), *LcMADS75* (*LcAP3-1*), *LcMADS45* (*LcAP3-2*), *LcMADS50* (*LcPI*), *LcMADS65* (*LcAG*), *LcMADS51* (*LcSTK*), *LcMADS11* (*LcSHP*), *LcMADS100* (*LcSEP1*), *LcMADS94* (*LcSEP2*), *LcMADS91* (*LcSEP3*), and *LcMADS73* (*LcSEP4*) represented ABCDE genes. A previous study observed the regulation of GA and its inhibitor (uniconazole) in litchi sex determination [17], and JA has shown an antagonistic role to GA in flower development [18]. Hence, we tested the response of genes involved in the ABCDE model to these two hormones in this study.

**Figure 9:**
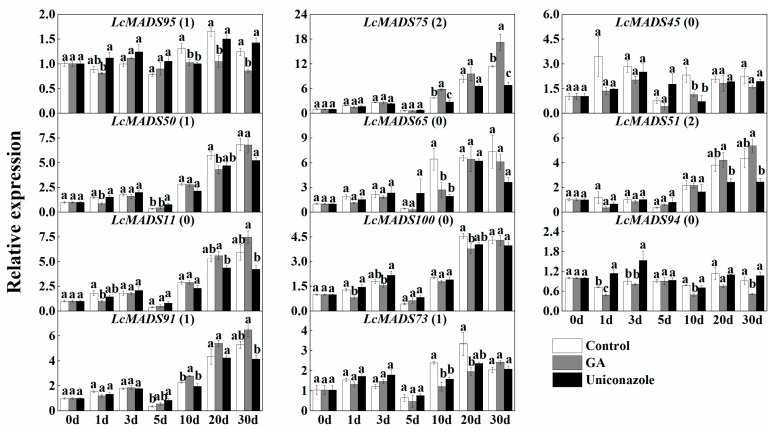
Relative expression of litchi MADS-box genes involved in the ABCDE model in response to treatments of GA or uniconazole. The number following the gene name indicates the number of predicted GA response elements in the promoter region. Data shown are mean ± SE of at least three biological replicates. Differences between treatments at each time point were analyzed by one-way ANOVA with Duncan’s post-hoc test. Significant differences between treatments at each time point are indicated with letters (*p* < 0.05).

The results showed that *LcMADS75* (*LcAP1*) was significantly up-regulated 10 d and 30 d after the GA treatment, but showed an opposite trend after the uniconazole treatment (Figure 9). A similar expression pattern in response to GA could be observed for *LcMADS51* (*LcSTK*), which had two GA response elements, just as *LcMADS75* did. However, other genes having one or no GA response elements did not show a significant response to GA or its inhibitor. These data suggest that the response of MADS-box genes to GA is consistent with the prediction of promoter elements.

**Figure 10:**
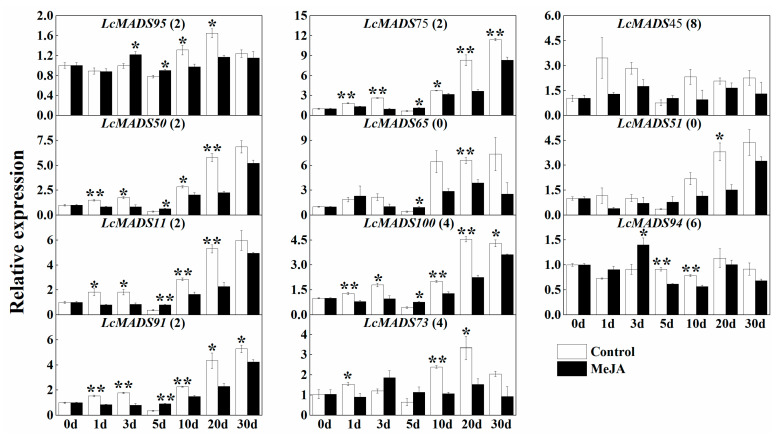
Relative expression of litchi MADS-box genes involved in the ABCDE model in response to treatment with MeJA. The number following the gene name indicates the number of predicted GA response elements in the promoter region. Data shown are mean ± SE of at least three biological replicates. Significant differences between treatments at each time point determined by an independent sample *t*-test are indicated with * (*p* < 0.05) and ** (*p* < 0.01).

The expression of putative ABCDE genes, except for *LcMADS94*, gradually increased as the litchi bloomed (Figure 10). After MeJA treatment, most genes were significantly down-regulated in litchi inflorescences, such as *LcMADS95*, *LcMADS75*, *LcMADS50*, *LcMADS11*, *LcMADS100,* and *LcMADS91*, indicating a negative correlation of JA in litchi flower development. Interestingly, *LcMADS45*, having eight JA response elements in its promoter region, did not significantly respond to MeJA at any development stage (Figure 10). 

## 3. Discussion

In plants, MADS-box transcription factors play important roles in flowering and floral organ development [19,20]. Therefore, the identification and evolutionary analysis of MADS-box families have been intensively studied in many species, such as *Arabidopsis thaliana* (107 genes), *Populus trichocarpa* (105 genes), *Pyrus bretschneideri* (95 genes), *Malus × domestica* (147 genes), and rice (75 genes) [8,21,22,23,24]. In comparison with these species, some plants, including pineapple (48 members) and bamboo (42 members), possess a relatively smaller number of MADS-box genes [25,26]. In the litchi genome, 101 MADS-box genes were identified in this study (Figure 1), similar to its Sapindaceae relative longan (*Dimocarpus longan*), which has 91 MADS-box members [27]. The difference in MADS-box gene numbers among species could be the result of genome duplications. For example, pineapple has experienced two ancient whole genome duplications, whereas rice has gone through a recent whole genome duplication, resulting in more MADS-box genes than pineapple [24,25,28]. The variation in members of the MADS-box family between species implicates divergence in regulatory mechanisms of flowering and flower development. 

Among type II genes, the MIKC^C^ cluster is well known for its plant-specific and important roles in floral organogenesis. However, flowering plants widely differ in the number of MIKC^C^ family genes. For example, this study found ten putative SVP members compared to two *AtSVP* paralogs in *Arabidopsis* (Figure 3), indicating that additional lineage-specific duplication events occurred in litchi for the SVP subfamily genes. SVP serves as a repressor of flowering time via suppressing *SOC1* transcription in response to ambient temperature and gibberellin [29,30]. As an evergreen species in the subtropics, litchi has flowering that is induced by cold temperatures and by GA inhibitors such as uniconazole, paclobutrazol, and daminozide [31,32]. During the cold-dependent floral induction in litchi, the expression of *LcSVP* homologs decreases in apical meristems and panicle primordia, and this decrease can be alleviated by brassinosteroid treatment [33]. SVP also interacts with other MADS-box members, such as AP1 and AGAMOUS-LIKE24 (AGL24), to repress floral homeotic genes controlling petal, stamen, and carpel identity [34]. For example, transgenic *Arabidopsis* over-expressing *AtSVP* orthologs from *Actinidia* spp. or barley (*Hordeum vulgare*) results in leaf-like sepals and petals [35,36]. Therefore, more putative SVP homologs in litchi might involve more complicated regulation of flowering time and flower development in comparison with *Arabidopsis*.

On the contrary, litchi has fewer FLC members compared to *Arabidopsis* [37]. In *Arabidopsis thaliana*, FLC encodes a floral repressor whose expression is epigenetically silenced by prolonged cold exposure in integrating the autonomous and vernalization flowering pathways [38,39]. The transcriptional silencing involves the cold-induced FLC antisense transcript, *COOLAIR*, whose accumulation causes a switch of the chromatin states at FLC [40,41]. FLC is a transcription factor that can directly repress the expression of *FT* (*FLOWERING LOCUS T*) to inhibit flowering [42]. However, the FLC clade has been demonstrated to be absent in plants that do not require vernalization for flowering, such as pineapple and rice [24,25]. This difference may explain why few FLC members are present in litchi. Although low temperatures under 20 °C are required for litchi floral induction, this cold requirement can be reduced or replaced by drought treatment, indicating that other players may compensate for FLC and participate in the cold-related flowering pathway in litchi [43]. In addition, FLC interacts with SVP in vivo to associate with the promoter of *SOC1* and *FT*, and their function is mutually dependent [30]. This interaction is critical for their function in determining flowering, because loss of function of either gene compromises the ability of the other gene to repress flowering. Therefore, a trade-off is suggested to exist between the number of SVP and FLC genes in plants to control vernalization/cold-dependent flowering. 

Promoter analysis of MADS-box genes in litchi identified numerous putative ABA and JA response elements, suggesting regulation by these hormones (Figure 8). The promotion of flowering and flower formation by ABA has been illustrated in litchi and apple [44,45], in contrast to *Phalaenopsis hybrida* and *Pharbitis nil*, in which ABA has been shown to inhibit flowering [46,47]. In addition, genes related to litchi flower sex determination were also suggested based on the transcriptome data derived from stamens and carpels. JA signaling has been shown to induce the elongation of anther filament, the opening of stomium at anthesis, and the production and release of viable pollen [48,49,50]. The male fertility of JA mutants can be restored by application of exogenous jasmonic acid [51]. In accordance with the prediction of cis-elements of MADS-box genes, the transcription of selected ABCDE genes increases as litchi flowers develop but decreases in response to exogenous treatment with JA (Figure 10). Therefore, JA might negatively contribute to litchi flower development, consistent with the conclusion in a maize study that JA suppresses pistil development [18]. 

*LcMADS51* (LcSTK), a putative D-class gene, has showed a higher expression level in the carpel of female flowers as opposed to other flower types; this expression increased during development of the female flowers (Figure 6 and Figure 7), indicating a positive role in carpel development in litchi [52]. Moreover, the expression of *LcMADS51* increased in response to GA treatment and significantly decreased in response to the inhibitor of GA biosynthesis (uniconazole), suggesting a positive role for GA in litchi carpel formation. This result is in agreement with previous observations in corn, *Sagittaria latifolia*, and *Jatropha curcas* [53,54,55]. GA can also promote the development of stamens and male fertility based on studies in *Arabidopsis*, cucumber, and spinach [56,57,58]. Thus, further studies are required to pinpoint the molecular basis of this species-dependent role of GA in sex determination.

## 4. Materials and Methods

### 4.1. Whole-Genome Identification of MADS-Box Genes

The MADS-box protein sequences of *Arabidopsis* and rice were obtained from TAIR (http://www.arabidopsis.org/, accessed on 23 March 2021) and RGAP (http://rice.plantbiology.msu.edu/, accessed on 23 March 2021) databases, respectively. These sequences were used as queries to search potential MADS-box genes by BLAST against the litchi genome with TBtools software (v1.09854; https://github.com/CJ-Chen/TBtools/releases, accessed on 23 March 2021) [59]. In addition, the MADS-box SRF family domain (PF00319) was used to identify the MADS-box proteins in the litchi genome using a Hidden Markov Model Search. All the predicted sequences were further validated using NCBI’s Conserved Domain Database (CDD) (http://www.ncbi.nlm.nih.gov/cdd/, accessed on 23 March 2021) and EMBL’s Simple Modular Architecture Research Tool (SMART, http://smart.embl-heidelberg.de/, accessed on 23 March 2021) [60] to search for conserved domains. Finally, all candidate MADS-box genes were manually examined to remove incomplete and redundant sequences.

### 4.2. Phylogenetic Analysis of MADS-Box Genes and Mapping on Chromosomes 

MADS-box genes in *Arabidopsis* and rice were used for the classification of litchi MADS-box genes. Multiple sequence alignment of the full-length protein sequences of AtMADSs, OsMADSs, and LcMADSs was performed using Muscle software with default parameters [61]. A maximum likelihood phylogenetic tree was constructed using MEGA 7.0 software with a bootstrap value of 1000 [62,63]. The missing data and gaps were processed by partial deletion. The *LcMADS*s were classified according to the phylogenetic relationships with MADS-box homologs in *Arabidopsis* and rice. The litchi genome has been mapped to 15 chromosomes. The physical locations of litchi MADS-box genes were mapped onto chromosomes using TBtools software [59].

### 4.3. Gene Domain, Structure, and Conserved Motif Analysis

Litchi MADS-box genes were analyzed for conserved domains using NCBI’s Batch CDD program [64]. TBtools was used to identify the gene structure based on the full-length coding sequences (CDS) and genomic sequences. Conserved motifs were analyzed using the Multiple Em for Motif Elicitation (MEME) online program (v5.3.3; http://meme-suite.org, accessed on 23 March 2021) with the following parameters: the number of repetitions was set to zero or one and the maximum number of motifs was 20 [65]. MADS-box genes were analyzed for motifs using the SMART program (http://smart.embl-heidelberg.de/, accessed on 23 March 2021) [46]. Gene domain, structure, and conserved motif were visualized with TBtools software.

### 4.4. Cis-Element Enrichment Analysis

Cis-regulatory elements within 2-kb upstream of the predicted translation start codon of litchi MADS-box genes were identified using the PlantCARE database tool (http://bioinformatics.psb.ugent.be/webtools/plantcare/html/, accessed on 23 March 2021) [66]. Here, we selected cis-elements associated with responses to phytohormones, including auxin, abscisic acid (ABA), ethylene, gibberellin (GA), methyl jasmonate (MeJA), and salicylic acid (SA).

### 4.5. Plant Materials

The ‘Feizixiao’ litchi plants used in this study were grown in an orchard located on the campus of South China Agricultural University (23° 9′ 50′’ N; 113° 21′ 20′’ E), Guangzhou, China. Carpels and stamens of the three types of litchi flowers (Figure S1), including full-bloom functionally female flowers with rudimentary stamens (female, F), functionally male flowers with rudimentary unobvious pistils (Male, M), and functionally male flowers with rudimentary obvious pistil (male, m), were collected separately. In addition, whole flowers were collected at five developmental stages based on size: 0.5–1 mm, 1–1.5 mm, 1.5–2 mm, half-bloom, and full bloom. 

The ‘Feizixiao’ litchi plants were sprayed with gibberellic acid (GA_3_, 100 mg/L), uniconazole (50 mg/L), and MeJA (1 mM) when the length of inflorescences was approximately 10 cm. Inflorescences were collected from both control and treated plants at 0 d, 1 d, 3 d, 5 d, 10 d, 20 d, and 30 d after treatment. All plant material was sampled in triplicate from north-, south-, east-, and west-facing parts of the tree in the morning (8 to 10 AM). The tissue was flash-frozen in liquid nitrogen and stored at −80 °C.

### 4.6. Analysis of Gene Expression by RNA-Sequencing and Quantitative Real-Time PCR

Total RNA was extracted using an RNA Extraction kit (Tiandz, Beijing, China) according to the manufacturer’s instructions. The samples were treated with DNase to remove residual genomic DNA. Both RNA-sequencing (RNA-seq) and quantitative real-time PCR (Q-PCR) methods were used to determine the expression of MADS-box genes. RNA-seq was conducted by Illumina sequencing at Biomarker Technologies Corporation (Beijing, China). The raw reads were trimmed and filtered with Trimmomatic software (v0.33) to remove adapters and low-quality reads. The high-quality reads were blasted against the sillva SSU and LSU ribosome RNA (rRNA) database, and the matched reads were removed to produce clean reads. The expression calculation at the transcript and gene level was conducted using Cufflinks (v2.2.1) with default parameters. The uniquely mapped reads were transformed into FPKM (fragments per kilobase of transcript per million mapped) values [67]. The RNA-seq datasets presented in this study were deposited in the Gene Expression Omnibus (GEO) database and are accessible through GEO code GSE182447 (https://www.ncbi.nlm.nih.gov/geo/query/acc.cgi?acc=GSE182447, accessed on 19 August 2021).

For qPCR analysis, 500 ng of total RNA was synthesized into cDNA using HiScript II reverse transcriptase (Vazyme Biotech, Nanjing, China). qPCR was performed according to the manufacturer’s specifications of SYBR Premix Ex Taq (TaKaRa Bio, Inc.) on a LightCycler 480 II (Roche, Germany). The gene expressions were normalized against a reference gene *LcActin* (HQ615689) [43,68]. Primers used in this study were designed on the website of Primer 3 (v 0.4.0; http://bioinfo.ut.ee/primer3-0.4.0/primer3/, accessed on 5 March 2020) and are shown in Table S3. Each expression profile was verified in three biological replicates. Relative expression level of each gene was calculated by the 2^-ΔΔCt^ method [69]. Gene expression profiles were visualized as a heat map via TBtools software [59].

### 4.7. Statistical Analysis

Differences between control and MeJA treated plants at each time point were investigated by an independent sample t-test. For two sample comparisons, significant differences are indicated with an asterisk symbol. Differences between control plants, and plants treated with GA and uniconazole at each time point, were analyzed by one-way analysis of variance (ANOVA) followed by a Duncan test using SPSS software (v.24, IBM). For multiple comparisons, significant differences are indicated with letters. Data shown are mean ± SE of at least three biological replicates.

## Data Availability

The RNA-seq datasets presented in this study were deposited in the Gene Expression Omnibus (GEO) database and are accessible through GEO code GSE182447 (https://www.ncbi.nlm.nih.gov/geo/query/acc.cgi?acc=GSE182447, accessed on 19 August 2021).

## Supplementary Materials

The following are available online at www.mdpi.com/article/10.3390/plants10102142/s1. Figure S1. The pictures of three types of litchi flowers and inflorescences. Figure S2. Litchi MADS-box protein domains. Green boxes indicate MADS domain, and yellow boxes indicate K domain. Figure S3. Expression profiles of litchi MADS-box genes in various floral organs. Heatmap result was done by column. Scaled log2 expression values are shown from blue to red, indicating low to high expression. M-carpel, the pistils in full-bloom functionally male flower (Male, M); M-stamen, the stamens in full-bloom functionally male flower (Male, M); F-carpel, the pistils in full-bloom functionally female flower (Female, F); F-stamen, the stamens in full-bloom functionally female flower (Female, F); m-carpel, the pistils in full-bloom functionally male flower (male, m); m-stamen, the stamens in full-bloom functionally male flower (male, m). Figure S4. Expression profiles of litchi MADS-box genes at five developmental stages of flower. Heatmap was created based on the FPKM values of litchi MADS-box genes from the transcriptome data. The normalization was done by column. Fl, 0.5-1 mcm female flower buds; F2, 1-1.5 mm female flower buds; F3, 1.5-2 mm female flower buds; F4, half-bloom female flowers; F5, full bloom female flowers; M1, 0.5-1 mm male flower buds; M2, 1-1.5 mm male flower buds; M3, 1.5-2 mm male flower buds; M4, half-bloom male flower; M5, full bloom male flowers. Figure S5. Expression confirmation of four BC class genes using q-PCR in litchi floral organ. Figure S6. Expression confirmation of four BC class genes using q-PCR at different female development stages. Figure S7. Expression confirmation of four BC class genes using q-PCR at different male development stages. Table S1. The MADS-box transcription factors identified in litchi. Table S2. Motif sequences identified within the litchi MADS-box genes using the MEME tool. Table S3. Primers used in this study. Table S4. Cis-elements within 2-kb upstream of transcription start codon of litchi MADS-box genes.
