# Supplementary material for: Genome-Wide Identification and Expression Analysis of MADS-Box Family Genes in Litchi (Litchi chinensis Sonn.) and Their Involvement in Floral Sex Determination
Source: Plants (Basel). 2021 Oct 9;10(10):2142. doi: 10.3390/plants10102142 (PMC8540616; doi:10.3390/plants10102142)
Supplement: Supplementary file 1 [file plants-10-02142-s001.zip › supplementary data/Legend to supplementary figures and tables.pdf]

Fig S1. The pictures of three types of litchi flowers and inflorescences.

Fig S2. Litchi MADS-box protein domains. Green boxes indicate MADS domain, and yellow boxes indicate K domain.

Fig S3. Expression profiles of litchi MADS-box genes in various floral organs. Heatmap result was done by column. Scaled log_2 expression values are shown from white to red, indicating low to high expression. M-carpel, the pistils in full-bloom functionally male flower (Male, M); M-stamen, the stamens in full-bloom functionally male flower (Male, M); F-carpel, the pistils in full-bloom functionally female flower (Female, F); F-stamen, the stamens in full-bloom functionally female flower (Female, F); m-carpel, the pistils in full-bloom functionally male flower (male, m); m-stamen, the stamens in full-bloom functionally male flower (male, m).

Fig S4. Expression profiles of litchi MADS-box genes at five developmental stages of flower. Heatmap was created based on the FPKM values of litchi MADS-box genes from the transcriptome data. The normalization was done by column. Fl, 0.5-1 cm female flower buds; F2, 1-1.5 cm female flower buds; F3, 1.5-2 cm female flower buds; F4, half-bloom female flowers; F5, full bloom female flowers; M1, 0.5-1 cm male flower buds; M2, 1-1.5 cm male flower buds; M3, 1.5-2 cm male flower buds; M4, half-bloom male flower; M5, full bloom male flowers.

Fig S5. Expression confirmation of four BC class genes using q-PCR in litchi floral organ.

Fig S6. Expression confirmation of four BC class genes using q-PCR at different female development stages.

Fig S7. Expression confirmation of four BC class genes using q-PCR at different male development stages.

Table S1. The MADS-box transcription factors identified in litchi.

Table S2. Motif sequences identified within the litchi MADS-box genes using the MEME tool.

Table S3. Primers used in this study.

Table S4. Cis-elements within 2-kb upstream of transcription start codon of litchi MADS-box genes.
