# Supplementary material for: Genome-Wide Identification and Expression Analysis of MADS-Box Family Genes in Litchi (Litchi chinensis Sonn.) and Their Involvement in Floral Sex Determination
Source: Plants (Basel). 2021 Oct 9;10(10):2142. doi: 10.3390/plants10102142 (PMC8540616; doi:10.3390/plants10102142)
Supplement: Supplementary file 1 [file plants-10-02142-s001.zip › supplementary data/Table S2.pdf]

Table S2 Motif sequences identified by MEME tools in litchi MADS-box genes.

**Motif**	**Length**	**Best possible match**
1	41	RQVTFSKRRNGLFKKASELSTLCDAEVALIVFSPTGKLYEF
2	15	MGRGKIEJKRIENET
3	24	RQLLGEDLQGLSIKELQQLEKQLE
4	28	RVRETKNZLIMEZISELKKKEQQLQEEN
5	50	SSSSTKQVIEKYKMNMPNLHRFDQPSLELQLECNTYAMLSREIAEKTREL
6	50	WPSQMGVQEVLSRFKNMPEMERSKKMVNQDSFLRZRIVKANEQLKKQRKD
7	21	GHPSIESVVNRFJTGNPPQDS
8	27	WEAPIDELNPEQLEELKAALEKLKKKV
9	29	AHRNANIQELNMZLTDLLNZLEIEKERGE
10	15	SSSSMEKTJERYQKC
11	21	QSWQQEYAKLKQQIZNLQRSQ
12	41	LHHIYQGNGFDALNVNDFHSLIWFAQEKRKEIKKRVDYLQQ
13	41	FASCWDDRLNSFSIDQLRVLLVTFDNNIEIARTRIATIKGD
14	50	NDIAIGLQQFGNFGTGGGGNASGQDMRLPTEFFDPNIMTSDIGLPYDVTK
15	41	GSHDNZGVIWTPDGGHHDGLIFDDEYPLNDLVGLDEIDYBP
16	29	QYLKDIEKKIETHNQMAAGGSSSSSSTAA
17	50	QMELVDYRNYPSYFGGFNGAETTMRLQPYEGFKGLMNIGGMNVPMLQPQP
18	15	NSSDTSLKLGLAFPS
19	48	LZEKVAEKKKILSEWKNPEKIDDLEKJEIMENHLAESLEBIKAKKEEL
20	29	EVELIRKENKETLKKLEEERKKNRGEKTG
