# Supplementary material for: Genome-Wide Identification and Expression Analysis of MADS-Box Family Genes in Litchi (Litchi chinensis Sonn.) and Their Involvement in Floral Sex Determination
Source: Plants (Basel). 2021 Oct 9;10(10):2142. doi: 10.3390/plants10102142 (PMC8540616; doi:10.3390/plants10102142)
Supplement: Supplementary file 1 [file plants-10-02142-s001.zip › supplementary data/Table S3.pdf]

Table S3 Primers used in this study.

ID	Name	Forward primer (5'-3')	Reverse primer (5'-3')
LITCHI005352.m1	LcMADS95(AP1)	CCCTAATGCACTCTCCTTCCT	CTGGGTTTCTTGTTTGCATTCA
LITCHI006530.m1	LcMADS75(AP3-1)	AGCCCTACCACAACTACGAA	TTCACCAATCCTCTGCCTGA
LITCHI010203.m1	LcMADS45(AP3-2)	TCTGTGAAGGTTGTTCGTGA	AGATGGGCAGCTGAGTTTAAA
LITCHI011302.m1	LcMADS50(PI)	GGGAGATGGCAATGGAGAAT	ATAGGCTGAACACGGAATGC
LITCHI028933.m1	LcMADS65(AG)	AGAATGAGCTGTTGTTTTCGG	TGCCGTATGTCCACTAAATGT
LITCHI011705.m1	LcMADS51(STK)	GGATTGCGGACATTGAGAGG	TGGAGCAGTTTCTTGTCAGGA
LITCHI026995.m1	LcMADS11(SHP)	ACACAAGTTGGAGAGCAGTC	GGCCAGTTTATCATGAGTCATCT
LITCHI019333.m4	LcMADS100(SEP1)	TGAGGGCTTACAACGAACTCA	AGGATGATTCTAGCTGACGC
LITCHI005351.m1	LcMADS94(SEP2)	GCCCTACAACGCTCACAAAG	TCCCCAATGAACTGTCTAGGT
LITCHI004634.m1	LcMADS91(SEP3)	CCAAGTGCAGAAGATGTGGG	GTTCACACTTGGACCGGC
LITCHI023328.m1	LcMADS73(SEP4)	TTGACCAGCTTGCGGATCTT	TAGGCAGCAAGAGAGTGGTC
LITCHI007623.m1	LcActin	ACCGTATGAGCAAGGAAATCACTG	TCGTCGTACTCACCCTTTGAAATC
