# Supplementary figures and images for: Genome-Wide Identification and Expression Analysis of MADS-Box Family Genes in Litchi (Litchi chinensis Sonn.) and Their Involvement in Floral Sex Determination
Source: Plants (Basel). 2021 Oct 9;10(10):2142. doi: 10.3390/plants10102142 (PMC8540616; doi:10.3390/plants10102142)

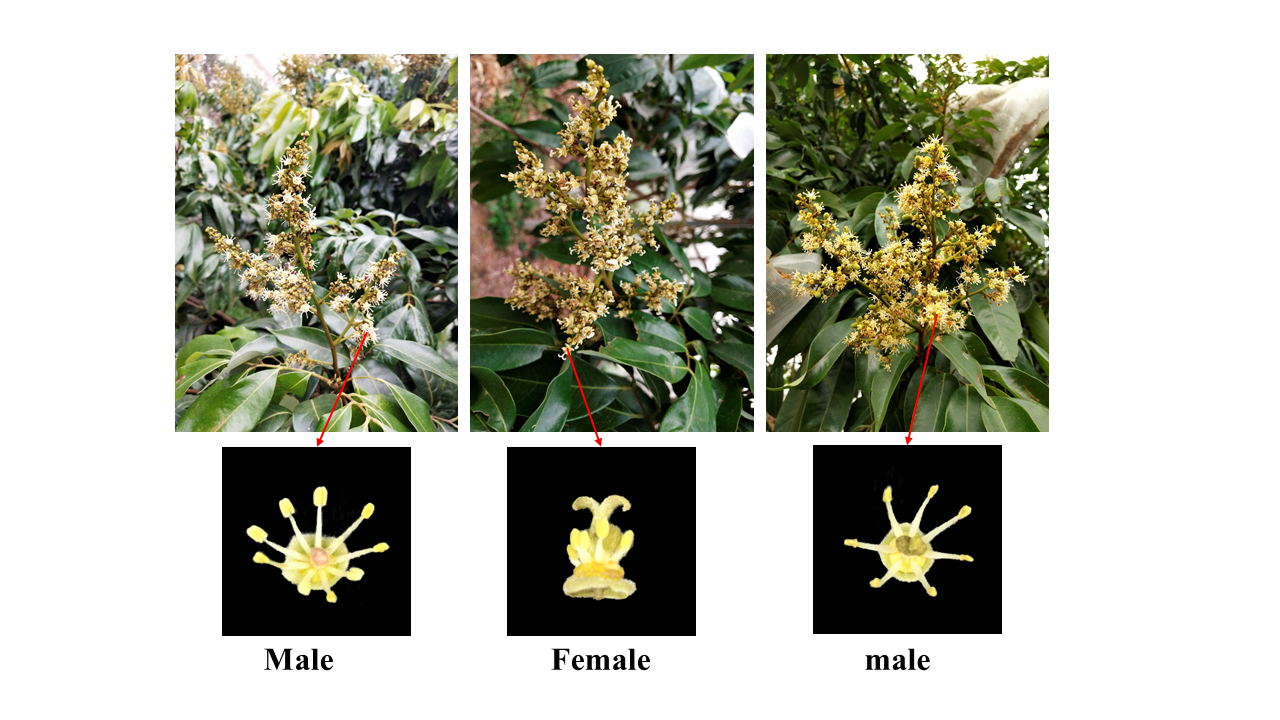

Supplement: Supplementary file 1 [file plants-10-02142-s001.zip › supplementary data/Fig S1.tif]

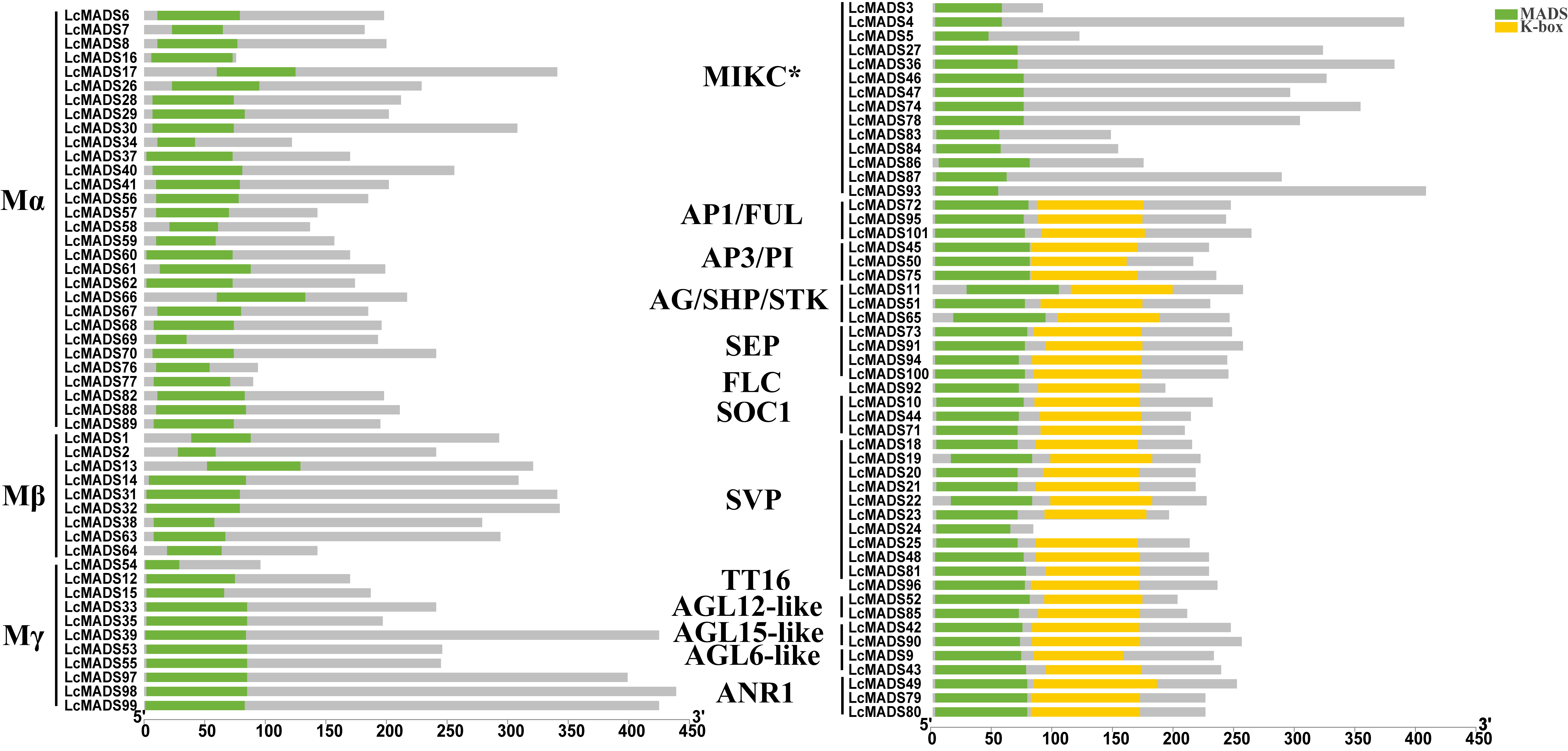

Supplement: Supplementary file 1 [file plants-10-02142-s001.zip › supplementary data/Fig S2.tif]

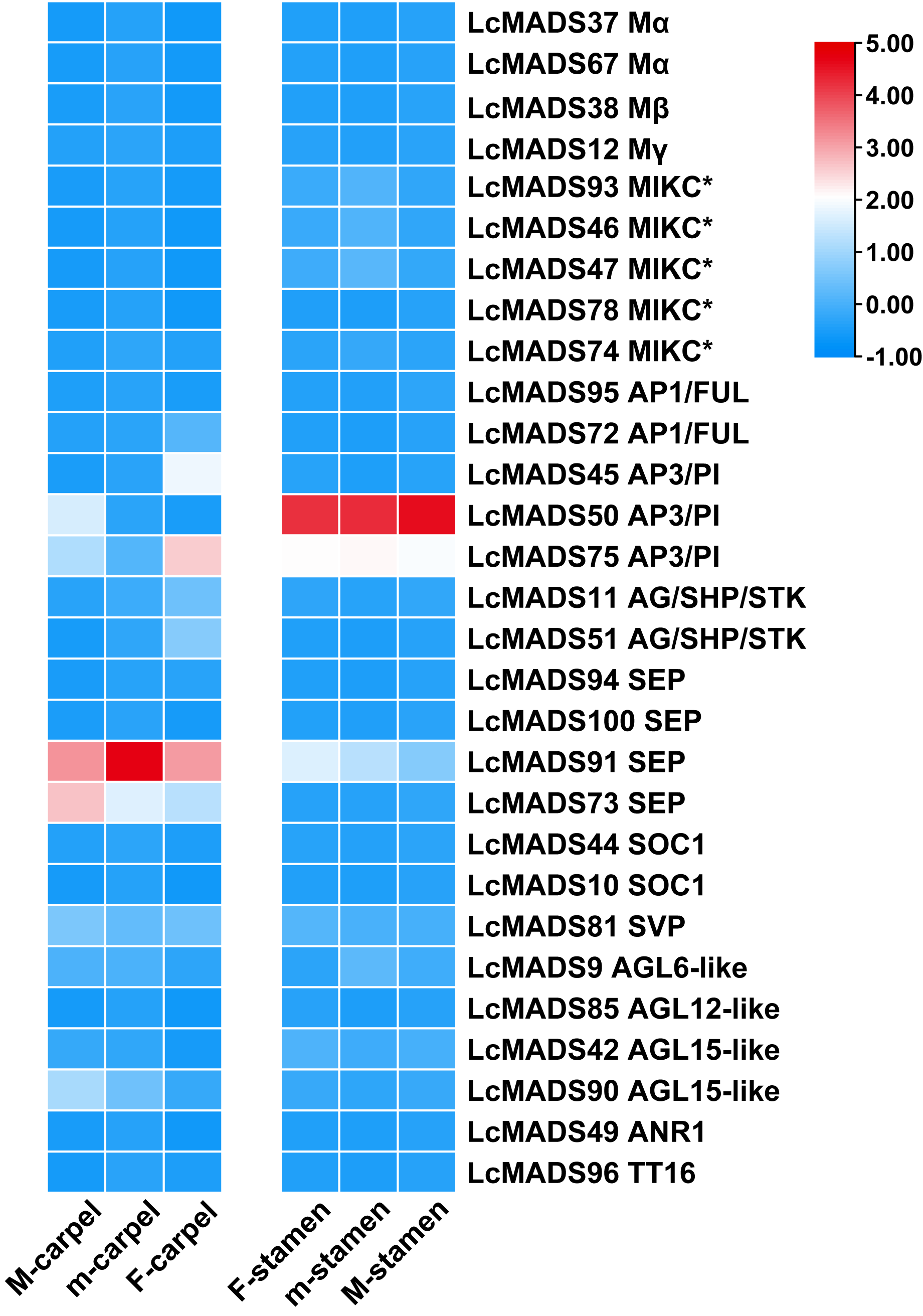

Supplement: Supplementary file 1 [file plants-10-02142-s001.zip › supplementary data/Fig S3.tif]

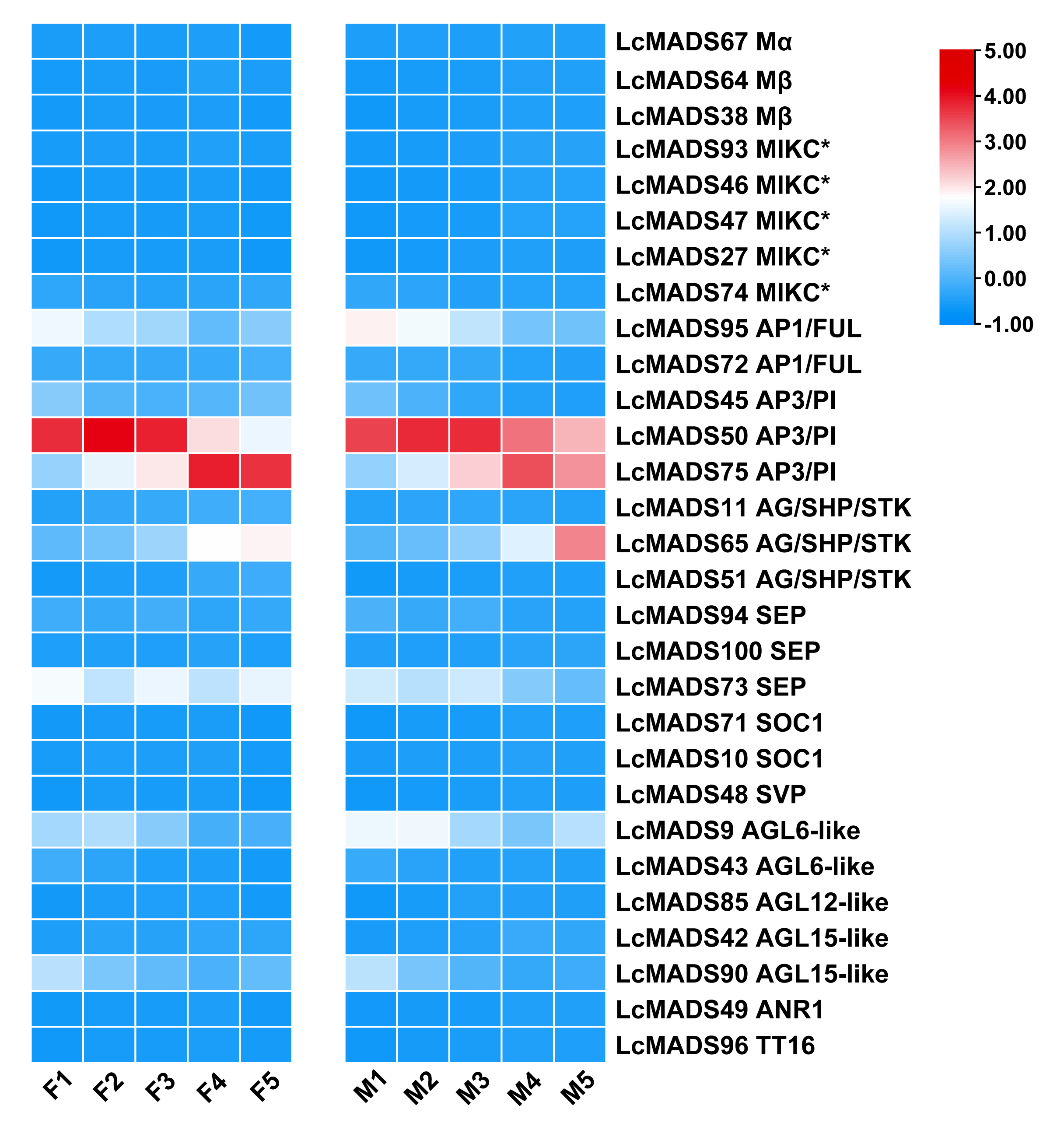

Supplement: Supplementary file 1 [file plants-10-02142-s001.zip › supplementary data/Fig S4.tif]
